# A challenging case of arrhythmogenic right ventricular cardiomyopathy presenting as fulminant myocarditis

**DOI:** 10.1093/omcr/omab013

**Published:** 2021-04-28

**Authors:** Mohammad Mahdavi, Leila Hosseini, Kambiz Mozzaffari, Fatemeh Zadehbagheri, Nahid Rezaeian

**Affiliations:** 1 Rajaie Cardiovascular Medical and Research Center, Iran University of Medical Sciences, Tehran, Iran; 2 Cardiology Department, North Khorasan University of Medical Sciences, Bojnurd, Iran; 3 Cardiology Department, Yasuj University of Medical Sciences, Yasuj, Iran

## Abstract

Arrhythmogenic right ventricular cardiomyopathy (ARVC) is known as a primary genetic heart disease that leading to the myocardial deposition of fibrofatty tissue in right ventricular (RV) wall. Sometimes, it occurs in the left ventricular (LV) subepicardial wall.

This study introduces a child referred to our hospital with influenza-like symptoms and ventricular tachyarrhythmia, followed by cardiac failure. However, in our subsequent evaluation, there was evidence of severe LV and RV dysfunction based on the echocardiography. Moreover, cardiac magnetic resonance showed not only the major criteria of ARVC but also those of Lake Luise seen in myocarditis. Regarding the deteriorating condition during the hospital course, he was later scheduled for heart transplantation.

Finally, the histopathological study of explanted heart revealed RV myocyte atrophy with the infiltration of fibrofatty tissue in myocardium diagnostic of ARVC, resolving dilemma between ARVC and myocarditis.

## INTRODUCTION

Arrhythmogenic right ventricular cardiomyopathy (ARVC) is an inherited disorder that leads to predominantly right ventricular (RV) dysfunction. Cardiac magnetic resonance (CMR) imaging is an excellent diagnostic modality in this disease, and the pathological findings also support it significantly. This case introduces a patient who had a combination of ARVC findings and myocarditis in CMR.

## CASE REPORT

A 9-year-old boy was admitted to pediatric ward with a history of flu-like symptoms of 1-week duration followed by abdominal pain.

He was on methylphenidate (10 mg/day) due to attention deficit hyperactivity disorder for 4 years; his past medical history was otherwise unremarkable.

In his family history, the sudden death of a sister at the age of 17 was considerable in which she had not been evaluated.

In the physical examination, he was afebrile with a 90/70 mm Hg blood pressure and an average cardiac rate of 135 beats/min. His oxygen saturation was 92% with no supplemental oxygen, and a respiratory rate of 30 breaths/min was noted.

Moreover, his heart rhythm was irregular due to frequent premature ventricular contractions (PVCs). Also, lung auscultation revealed diminished basal sounds, and abdomen bulged with positive shifting dullness but no peripheral edema. Regarding this finding, abdominal sonography was performed which indicated the significant ascites. Consequently, an abdominal tap was conducted that showed a transudative fluid.

**Figure 1:**
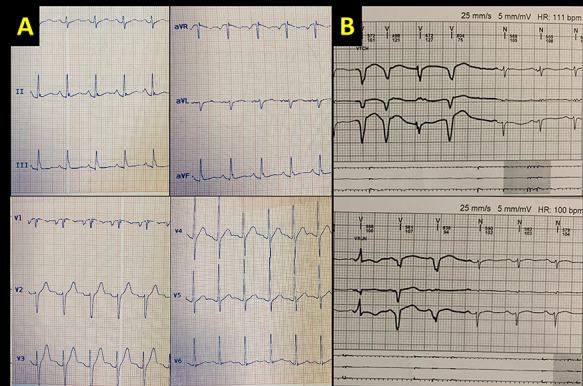
(**A**) The 12-lead ECG shows slight sinus tachycardia, epsilon wave, and T wave inversion in V1 and (**B**) Holter monitoring shows frequent premature ventricular complexes.

The lab data revealed an elevated cardiac troponin I (~1300 ng/ml), pro-brain natriuretic peptide (15 000 pg/ml) and C-reactive protein was (22 mg/l). His erythrocyte sedimentation rate was 56 mm/h. The electrolytes were normal, and so was his complete blood count. Bilateral pleural effusion was observed in chest X-ray. The evidence of sinus tachycardia, epsilon wave and bigeminy PVCs were found, but no other remarkable findings were detected on electrocardiography (ECG) (Fig. 1A). Besides, transthoracic echocardiography illustrated a reduced left ventricular (LV) ejection fraction (EF) (~20%) coupled with severe RV systolic dysfunction. Furthermore, color Doppler echocardiography revealed a severe regurgitation of mitral and tricuspid valves.

**Figure 2:**
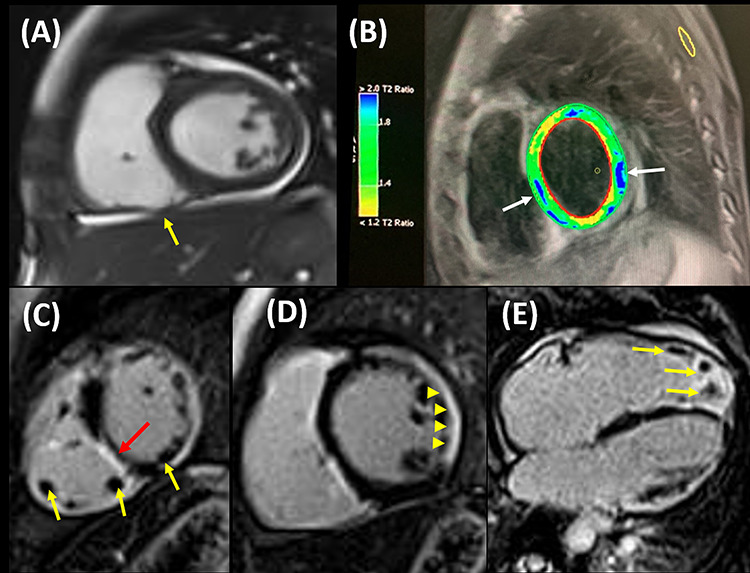
(**A**) B-SSFP image in mid short axis view shows the RV regional microaneurysm formation (yellow arrow), (**B**) STIR image reveals the regional myocardial edema (blue areas), (**C**) LGE image in SAX view indicates RV and LV apical clots (yellow arrow) and RV fibrosis (red arrow), (**D**) LGE image in mid-SAX view illustrates LV lateral wall subepicardial fibrosis (yellow arrowhead) and (**E**) LGE in axial view shows the multiple RV apical clots. B-SSFP: balanced steady-state free prerecession, SAX: short axis, STIR: short tau inversion recovery.

CMR revealed a dyssynchronous RV contraction, regional microaneurysm formation, as well as RV wall thinning. A significant decrease was also observed in RV EF (15%) and RV end-diastolic volume index (120 cc/m^2^), which met the major criteria of ARVC. Biventricular apical clot, active myocardial edema and fibrosis of base and mid-LV lateral wall were also identified (Fig. 2). Based on CMR data, a precise differentiation was challenging between active myocarditis and acute phase of ARVC.

During hospital course, repetitive ventricular tachyarrhythmia episodes occurred (Fig. 1B), for which amiodarone and lidocaine were prescribed to suppress ventricular arrhythmia, and intravenous immunoglobulin with corticosteroid was administered with suspicion of possible myocarditis.

**Figure 3:**
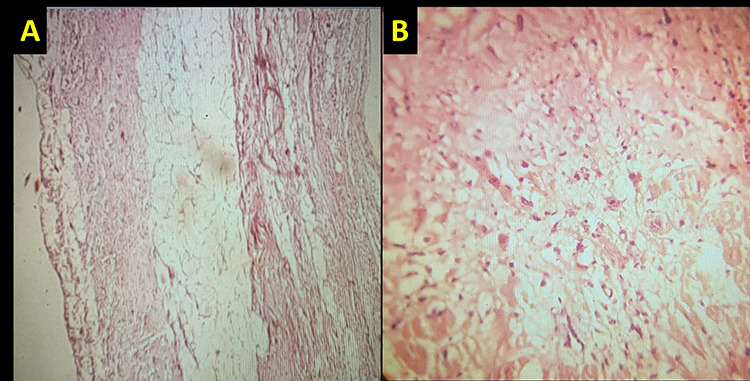
(**A**) RV specimen indicates the evidence of RV free wall fat replacement and adjacent myocardial fiber atrophy and (**B**) LV specimen revealed focal epicardial fibrosis and scant inflammatory cell infiltration with focal myocytolysis.

Nevertheless, because of refractory ventricular arrhythmia and New York Heart Association (NYHA) functional class (FC) IV, successful heart transplantation was done after 1 month despite full medical treatment. Besides, examining removed heart at transplantation was done. Moreover, in the gross pathologic specimen, RV had a thin, papered appearance. In the histologic specimens at LV lateral and septal wall, as well as RV free wall, there was evidence of fat replacement in RV free wall accompanied by myocardial fiber atrophy. Moreover, LV sections showed focal epicardial fibrosis with scant inflammation and focal myocytolysis (Fig. 3).

During a 14-month follow-up after the heart transplantation, the patient had NYHA FC = I. Endomyocardial biopsy showed no evidence of rejection, and heart catheterization revealed that RV pressure was ~20/6, mean pulmonary artery pressure = 12 mmHg, LV end-diastolic pressure of 10 mmHg and aortic pressure was ~105/60 mmHg. Moreover, the echocardiography data documented a preserved biventricular performance (LV EF = 50%, aortic velocity-time index = 16 cm, E/E′ = 10, RV ‘tricuspid annular plane systolic excursion’ = 18 cm and systolic tissue velocity of tricuspid valve annulus = 9 cm/s) with no more than mild valvular regurgitation.

## DISCUSSION

This patient was a challenging case with several findings in favor of active myocarditis versus an acute phase ARVC or a combination of both.

In this regard, the acute clinical picture of disease with a rise in cardiac biomarker levels, LV involvement, and CMR tissue characterization findings supported the diagnosis of active myocarditis. In contrast, RV regional microaneurysm formation, severely diminished RV systolic performance in CMR coupled with RV myocardial atrophy and fibrofatty replacement in the histologic specimens were in favor of ARVC. In a case series including six pediatric patients with a genetic diagnosis of ARVC who presented by myocarditis-like symptoms, active inflammation in myocardium compatible with acute myocarditis was seen in CMR in all the cases; however, it was without any evidence of documented infection preceding this episode, except in one patient [1].

In previous case, three main hypotheses can be concluded based on clinical, imaging, histology, and lab data.

1. Myocarditis superimposed on ARVC: The first hypothesis states that diseased myocardium in ARVC is more susceptible to a viral infection which results in the myocardial inflammation superimposed on ARVC [2].
2. Cardiotropic viruses with consequent ARVC: The second hypothesis assumes some viruses such as enterovirus and adenovirus can activate the mediators that damage the cardiac adherent junctions. Moreover, this process results in the irritability of ventricular myocardium with or without ion channel disruption and consequent ARVC symptomatology as a result of inflammation [3].

These cardiotropic viruses involve both ventricular myocardia with the complex cellular and adherent junction interaction, resulting in RV and LV inflammation and apoptosis that eventually induces myocardial fibrosis and remodeling [4, 5]. Moreover, in a case report study by Tanawuttiwat *et al*. [6], they assumed that there was a likelihood of insult from myocarditis by acquiring a desmosomal injury that eventually led to the anatomic and clinical presentations of ARVC.

1. The active phase of ARVC: The third hypothesis reinforces the concept that these myocarditis-like episodes are active phases of ARVC itself. In this line, Duarte Martins *et al*. indicated the data which ARVC could manifest as recurrent myocarditis-like episodes in the presence of myocardial inflammation evidence in CMR without an infectious trigger. They hypothesized that this presentation was an ‘active hot phase’ of disease course which might lead to disease progression [1].

We suppose that, in our case with preceding flu-like symptoms coupled with the significant LV involvement and cardiac biomarkers rise, the first and also the second hypotheses are at the top of list. However, we cannot completely rule out the acute phase of full-blown biventricular failure due to arrhythmogenic cardiomyopathy, especially when a family history of sudden death exists. Unfortunately, the genetic and molecular analyses were not performed for this patient and his family, due to the high price of the test.

Because of our case and another rare case report that has shown the relationship between acute myocarditis and ARVC, we suggest a comprehensive assessment of RV by cardiac imaging accompanied with the genetic test in the case of acute myocarditis to rule out ARVC, especially when the representing symptom is a cardiac arrhythmia [7].

## References

[1] Martins D , Ovaert C, Khraiche D, Boddaert N, Bonnet D, Raimondi F. Myocardial inflammation detected by cardiac MRI in arrhythmogenic right ventricular cardiomyopathy: a paediatric case series. Int J Cardiol 2018;271:81–6. 29885824 10.1016/j.ijcard.2018.05.116

[2] Sabel K-G , Blomström-Lundqvist C, Olsson SB, Eneström S. Arrhythmogenic right ventricular dysplasia in brother and sister: is it related to myocarditis? Pediatr Cardiol 1990;11:113–6. 2140890 10.1007/BF02239576

[3] Que D , Yang P, Song X, Liu L. Traditional vs. genetic pathogenesis of arrhythmogenic right ventricular cardiomyopathy. Ep Eur 2015;17:1770–6. 10.1093/europace/euv042 25921558

[4] Pieroni M , Dello RA, Marzo F, et al. High prevalence of myocarditis mimicking arrhythmogenic right ventricular cardiomyopathy: differential diagnosis by electroanatomic mapping-guided endomyocardial biopsy. J Am Coll Cardiol 2009;53:681–9. 19232901 10.1016/j.jacc.2008.11.017

[5] Patrianakos AP , Protonotarios N, Nyktari E, et al. Arrhythmogenic right ventricular cardiomyopathy/dysplasia and troponin release. Myocarditis or the “hot phase” of the disease? Int J Cardiol 2012;157:e26–8. 21962611 10.1016/j.ijcard.2011.09.017

[6] Tanawuttiwat T , Sager SJ, Hare JM, Myerburg RJ. Myocarditis and ARVC/D: variants or mimics? Hear Rhythm 2013;10:1544–8. 10.1016/j.hrthm.2013.06.008 23773988

[7] Ponsiglione A , Puglia M, Morisco C, et al. A unique association of arrhythmogenic right ventricular dysplasia and acute myocarditis, as assessed by cardiac MRI: a case report. BMC Cardiovasc Disord 2016;16:230. 27871237 10.1186/s12872-016-0412-2 PMC5117697

